# Knowledge and Awareness Regarding Osteoarthritis and Its Factors in Hail Region, Saudi Arabia

**DOI:** 10.7759/cureus.36557

**Published:** 2023-03-22

**Authors:** Shog K Alahmed, Ahmed M Mohyeldin, Areeb Alshammari, Ziyad F Alshammari, Ruba A Alhamdi, Sulaiman A Alghaslan, Hamad F Alshammari, Fahad F Alshamry, Ashwaq H Alshammari, Muath S Alhamdi

**Affiliations:** 1 Department of Surgery, University of Hail College of Medicine, Hail, SAU; 2 Medicine and Surgery, University of Tabuk, Tabuk, SAU

**Keywords:** saudi arabia, risk factors, osteoarthritis, joint disease, awareness

## Abstract

Objectives

Osteoarthritis (OA) is a common chronic degenerative joint disease linked to age, joint overuse abnormalities, and previous trauma. This research aims to assess the awareness levels, as well as the knowledge gap and misconceptions, about OA and its risk factors among the general population in Hail, Saudi Arabia.

Methods

The research adopted an observational cross-sectional method. Participants from Hail, Saudi Arabia, were recruited and then interviewed between 1 April and 15 July 2022. Adult males and females aged 18 or more were recruited via an online questionnaire using a Google Form link, inviting them to take part in a study concerning their knowledge of OA. The questionnaire was split into three sections. The first section covered demographic data, the second section contained general knowledge regarding OA, and the third section was made up of a 20-item quiz. The collected data was reviewed and then analyzed using the Statistical Package for Social Sciences (SPSS) Version 21 (IBM Corp., Armonk, NY, USA). The statistical methods employed were all two-tailed, with an alpha level of 0.05 considered significant if the P value was less than, or equal to, 0.05.

Results

Nine hundred six (906) eligible respondents completed the questionnaire. Participants ranged from 18 to 65 in age. More than 66% were female, while 77.5% had a university level of education or above. 13.6% had been diagnosed with OA. Overall, 40.9% of the study participants demonstrated a good knowledge level regarding OA, while 59.1% showed a poor knowledge level.

Conclusion

The study revealed that the awareness and knowledge levels of the general population in Hail about OA are unsatisfactory. Efforts are recommended to increase the awareness and knowledge of the population through public education, which in turn can lead to a reduction in risk factors and improved early detection of the disease.

## Introduction

Osteoarthritis (OA) is a joint disease that causes a person’s bones to rub against each other due to degeneration of the cartilage lining them [1]. Progressive loss and destruction of articular cartilage, osteophyte formation, subchondral bone thickening, variable degrees of synovium inflammation, knee menisci, and ligament degeneration, along with joint capsule hypertrophy, are pathological changes in OA joints [2]. The hallmark symptom and most common presenting complaint in persons with OA is joint pain [3]. However, there is a poor correlation between the radiographic changes and the degree of joint pain [4]. As OA is presently incurable, the primary goals of therapies are therefore to ease pain and improve joint function [5]. In the advanced stages, the patient may require invasive procedures, like joint replacement therapy [6].

Multiple risk factors are linked to the development of OA, with age being the primary risk factor [7]. The Framingham Study showed there was radiographic evidence of knee OA in 27% of the local population aged 63 to 70, rising to 44% in the over-80 age group [8]. Other risk factors include gender, genetic susceptibility, high body mass index (BMI), joint misalignment, abnormal joint shape, prior injury to the joint, and occupational and physical activities [9-10]. Regarding gender, females are more susceptible to OA [11]. Moreover, lower limb OA is more common in persons who have more physical occupations [12].

Worldwide, the burden of OA is growing. An estimated 28% of people over 60 suffer from the condition [13]. A meta-analysis published in 2021 reported that the prevalence of OA among individuals living in the Gulf Cooperation Council (GCC) countries was high at approximately 16.13% [14]. OA has been found to be more prevalent in Gulf Cooperation Council nations and Asian countries than in other nations in the world [14]. According to a cross-sectional study on the prevalence of knee osteoarthritis in Saudi Arabia among patients presenting to primary care with various medical complaints, 53.30% of male patients and 60.90% of female patients were reported to have knee osteoarthritis [15]. The 2017 Global Burden of Disease (GBD) study rated hip and knee OA as the 23rd leading cause of disability-adjusted life years and the 11th leading cause of disability worldwide [16]. In France, OA was found to be the leading cause of restrictions in activities, contributing to 22% of walking issues, 18.6% of carrying difficulties, and 12.8% of clothing difficulties. OA also contributed to the requirement for humanitarian aid. In addition, compared with the general population, patients with OA have an increased mortality risk [17]. In France, OA also had direct expenses of more than 1.6 billion euros in 2002, with hospital admissions accounting for more than half that total. Thirteen million hospital appointments for OA patients, in turn, resulted in 570 million euros in prescription drug costs [18]. Moreover, due to both the obesity epidemic and an increasingly elderly population, rates of persons with symptomatic OA are predicted to increase [19].

The aim of this research was to assess the awareness and knowledge levels, as well as the knowledge gap and misconceptions regarding OA and its risk factors among the general population in Hail, Saudi Arabia. Due to the increase in the prevalence of OA, recent guidelines for OA management should be considered in the GCC countries [14]. Weight loss for overweight or obese people, exercise, and walking aids for lower extremity osteoarthritis are among the most recent guidelines for non-pharmacological management of people with osteoarthritis [14]. The justification for selecting Hail is that there has been no discussion of the state of the field with respect to OA research in the Kingdom of Saudi Arabia (KSA).

## Materials and methods

Sample and setting

The study was conducted in Hail, Saudi Arabia, between April 1 and July 15, 2022. An observational cross-sectional design was employed. This study has been reviewed and approved by the Research Ethics Committee (REC) at the University of Hail, No: H-2022-421, dated December 19, 2022. A total of 384 participants were needed to estimate knowledge and awareness regarding osteoarthritis and its risk factors. The estimated sample size for this cross-sectional study was calculated using the following formula: ss=(Z2pq)/c2. Where ss = sample size, Z = 1.96, p = 0.5, q = (1-p) = 0.5, and c = sampling error of 5%. In total, 906 respondents participated. Incomplete submissions were excluded. Adult males and females aged 18 or more were recruited via an online questionnaire using a Google Form link, inviting them to take part in a study concerning their knowledge of OA. The agreement to fill out the questionnaire was considered consent to participate in the study. The invitation was sent using multiple social media platforms, i.e., WhatsApp, Twitter, Facebook, etc. Snowball sampling was utilized by encouraging participants to extend an invitation to more people using the same link they received.

Questionnaire development 

The questionnaire was developed by adopting multiple questions from other studies' validated questionnaires. In the end, the questionnaire was passed on to two orthopedic surgeons to ensure the accuracy of the items. A pilot study followed, and all items were understood by the participants. Thus, no further modifications to the questionnaire were made. The questionnaire was split into three sections. The first section covered demographic data, the second section contained general knowledge regarding OA, and the third section was made up of a 20-item quiz. The questions for the second section were taken verbatim from Alyami et al. (2020).

Statistical analysis

Data were collected, reviewed, and then analyzed via the Statistical Package for Social Sciences (SPSS) Version 21 (IBM Corp., Armonk, NY, USA). The employed statistical methods were all two-tailed, with an alpha level of 0.05 considered significant if the P value was less than, or equal to 0.05. Each correct answer received a score of 1 point. Participant knowledge about chronic OA was determined by tallying the discrete scores for the different questionnaire items. A total score of 60% was taken to signify a good level of awareness, while scores of less than 60% were considered poor. Descriptive analysis was conducted by prescribing frequency distributions and percentages for all study variables, including participants’ personal data and OA-related medical history, as well as whether they knew others with OA. Participants’ awareness about chronic OA was tabulated, whereas the overall awareness level was graphed. Cross-tabulation helped demonstrate the distribution of participants’ overall awareness levels, per their data and other factors. A Pearson chi-square test was used to determine significance, along with an exact probability test if there were small frequency distributions.

## Results

Nine hundred six (906) respondents completed the questionnaire. They were aged from 18 to 65 years old. The mean age was 34.2 ± 14.7. Six hundred four (604, 66.7%) participants were female. Seven hundred two (702, 77.5%) had a university education (or higher), while 175 (19.3%) had only a secondary level of education. One hundred twenty-three (123, 13.6%) had been diagnosed with OA. As for the mechanism of OA development, 237 (26.2%) believed that the cartilage at the end of the bones wears away over time, 119 (13.1%) thought that the nerve that passes near the joint is pressed, while 83 (9.2%) thought the amount of blood that reaches the joint decreases with age. Three hundred sixty-five (365, 40.3%) reported that they know someone with OA. The results are presented in Table 1.

**Table 1:** Bio-demographic data of study participants, Hail Region, Saudi Arabia

Bio-demographic data	No	%
Age in years		
18-25	297	32.8%
26-35	121	13.4%
36-45	226	24.9%
46-55	208	23.0%
56+	54	6.0%
Gender		
Male	302	33.3%
Female	604	66.7%
Education		
Below secondary	29	3.2%
Secondary	175	19.3%
University / above	702	77.5%
Have you been diagnosed with osteoarthritis?		
Yes	123	13.6%
No	783	86.4%
Do you know how osteoarthritis develops?		
The amount of blood that reaches the joint decreases with age	83	9.2%
The nerve that passes near the joint is pressed	119	13.1%
The cartilage at the ends of the bones wears away over time	237	26.2%
Accumulation of acids inside the joint	64	7.1%
I don't know	403	44.5%
Do you know anyone with osteoarthritis?		
Yes	365	40.3%
No	541	59.7%

66.9% of the participants answered that OA can impact different joints. 64.9% believe OA is a chronic issue. Only 15.7% thought osteoarthritis is rare. Regarding the causes of OA, 48.6% cited cold and damp weather, while 15% thought it resulted from a microorganism. As for the clinical presentation of OA, 60.4% stated that swelling is an indication of potential OA. 54.3% identified stiffness as an OA symptom. 34.8% said that pain is the only OA symptom. 65.7% said it could cause reduced joint movement. With respect to risk factors, 82.9% identified weight gain, 70.1% aging, and 44.9% genetic factors. 40.8% think men and women are equally affected. Regarding OA diagnostic methods, 58.5% cited physical examination and x-ray, while 37.4% highlighted blood tests. Considering management methods, 67.8% agreed that physiotherapy may result in OA symptom improvement. 52.2% agreed that some exercise, such as swimming, is suitable for OA sufferers. 47.2% reported that intra-articular injection of stem cells or hyaluronic acid is effective as a method to cure OA. However, 50.4% said joint replacement surgery is the ultimate solution for symptom relief. The results are presented in Table 2.

**Table 2:** Participant's knowledge and awareness regarding osteoarthritis (OA), Hail Region, Saudi Arabia

Knowledge and awareness items	Yes		No		I don't know	
	No	%	No	%	No	%
General knowledge						
Do you think osteoarthritis is a chronic problem?	588	64.9%	151	16.7%	167	18.4%
Do you think osteoarthritis is rare?	142	15.7%	573	63.2%	191	21.1%
Do you think that different joints can be affected by osteoarthritis?	606	66.9%	95	10.5%	205	22.6%
Causes of OA						
Do you think osteoarthritis is caused by cold, damp weather?	440	48.6%	233	25.7%	233	25.7%
Do you think it is developed by a microorganism?	136	15.0%	411	45.4%	359	39.6%
Clinical presentation of OA						
Do you think pain is the only symptom of osteoarthritis?	315	34.8%	371	40.9%	220	24.3%
Do you think stiffness is a symptom of osteoarthritis?	492	54.3%	127	14.0%	287	31.7%
Do you think swelling is a sign of osteoarthritis?	547	60.4%	145	16.0%	214	23.6%
Do you think osteoarthritis can lead to loss of joint movement?	595	65.7%	94	10.4%	217	24.0%
Risk factors of OA						
Do you think there are genetic factors that can predispose parson to osteoarthritis?	407	44.9%	255	28.1%	244	26.9%
Do you think aging is a risk factor for osteoarthritis?	635	70.1%	120	13.2%	151	16.7%
Do you think men and women are equally affected by osteoarthritis?	370	40.8%	324	35.8%	212	23.4%
Do you think weight gain is directly related to osteoarthritis?	751	82.9%	155	17.1%	0	0.0%
Diagnostic methods of OA						
Do you think physical examination and x-ray are used to diagnose osteoarthritis?	530	58.5%	117	12.9%	259	28.6%
Do you think blood tests are used to diagnose osteoarthritis?	339	37.4%	242	26.7%	325	35.9%
Management methods of OA						
Do you think NSAIDs can improve osteoarthritis symptoms?	357	39.4%	129	14.2%	420	46.4%
Do you think some forms of exercise like swimming is suitable for people with osteoarthritis?	473	52.2%	164	18.1%	269	29.7%
Do you think acid-free diets are a proven treatment for osteoarthritis?	308	34.0%	177	19.5%	421	46.5%
Do you think physiotherapy can cause a great improvement in the symptoms of osteoarthritis?	614	67.8%	120	13.2%	172	19.0%
Do you think intra-articular injection by stem cells or hyaluronic acid is an effective modality for curing OA?	428	47.2%	145	16.0%	333	36.8%
Do you think a joint replacement surgery will be the ultimate option to relieve the symptoms of osteoarthritis?	457	50.4%	173	19.1%	276	30.5%

**Figure 1:**
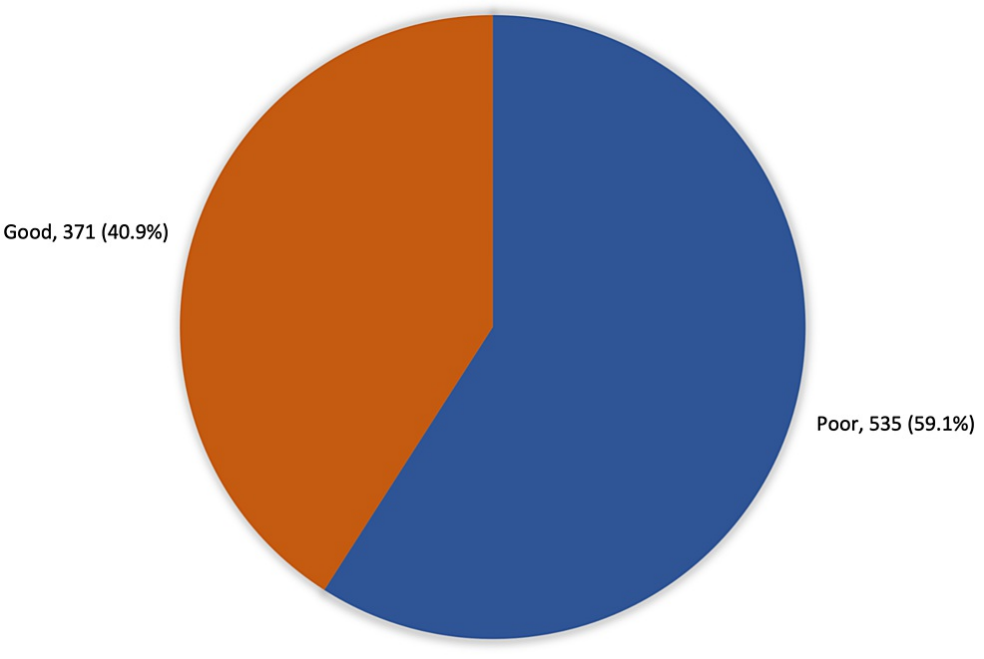
Overall knowledge and awareness regarding chronic osteoarthritis (OA) in Hail Region, Saudi Arabia

**Figure 2:**
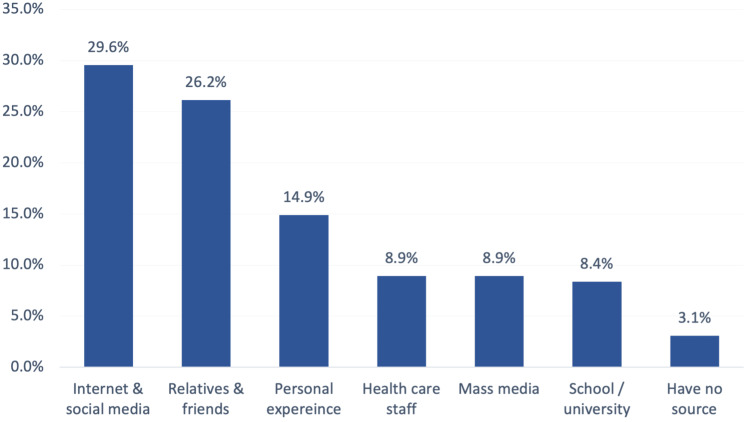
Source of information about chronic osteoarthritis (OA) in Hail Region, Saudi Arabia

Figure 1 details the overall levels of participant knowledge and awareness regarding chronic OA. Three hundred seventy-one (371, 40.9%) study participants showed a good knowledge level regarding OA, while 535 (59.1%) demonstrated a poor level of knowledge. In turn, Figure 2 details where participants found information about chronic OA. The most cited sources were the internet and social media (29.6%), followed by relatives and friends (26.2%), personal experience (14.9%), health care staff (8.9%), and mass media (8.9%).

Table 3 details the factors found to be associated with participant knowledge of chronic OA. 43.2% of university or above-educated participants demonstrated a good level of knowledge about OA, compared with 24.1% of those with a lower level of education. The recorded statistical significance was P=.024. Meanwhile, 57.8% of those who thought OA developed through the accumulation of acids inside the joint demonstrated good knowledge levels, compared with 29% of those who did not know (P=.001). Good knowledge levels were found among 58% of participants whose information came from health care staff, in comparison with 21.4% of those who had no specific source of information (P=.003).

**Table 3:** Factors associated with participants’ knowledge/awareness of chronic osteoarthritis (OA) P: Pearson X2 test $: Exact probability * P < 0.05 (significant)

Factors	Knowledge level				p-value
	Poor		Good		
	No	%	No	%	
Age in years					.185
18-25	174	58.6%	123	41.4%	
26-35	61	50.4%	60	49.6%	
36-45	137	60.6%	89	39.4%	
46-55	126	60.6%	82	39.4%	
56+	37	68.5%	17	31.5%	
Gender					.504
Male	183	60.6%	119	39.4%	
Female	352	58.3%	252	41.7%	
Education					.024*
Below secondary	22	75.9%	7	24.1%	
Secondary	114	65.1%	61	34.9%	
University / above	399	56.8%	303	43.2%	
Have you been diagnosed with osteoarthritis?					.474
Yes	69	56.1%	54	43.9%	
No	466	59.5%	317	40.5%	
Do you know how osteoarthritis develop?					.001*
The amount of blood that reaches the joint decreases with age	44	53.0%	39	47.0%	
The nerve that passes near the joint is pressed	74	62.2%	45	37.8%	
The cartilage at the ends of the bones wears away over time	104	43.9%	133	56.1%	
Accumulation of acids inside the joint	27	42.2%	37	57.8%	
I don't know	286	71.0%	117	29.0%	
Do you know anyone with osteoarthritis?					.240^$^
Yes	207	56.7%	158	43.3%	
No	328	60.6%	213	39.4%	
Source of information					.003*
Personal experience	80	59.3%	55	40.7%	
Relatives & friends	149	62.9%	88	37.1%	
Internet & social media	159	59.3%	109	40.7%	
Health care staff	34	42.0%	47	58.0%	
School / university	38	50.0%	38	50.0%	
Mass media	53	65.4%	28	34.6%	
Have no source	22	78.6%	6	21.4%	

## Discussion

This study analyzed the awareness levels and knowledge gap about OA and its risk factors present in the population of Hail, Saudi Arabia. Most participants were female (66.7%). The majority (77.5%) had a bachelor’s degree or above. The study found that of 906 participants, 371 (40.9%) had a good knowledge level regarding OA aspects such as risk factors, causes, symptoms, and management. A majority of 535 (59.1%) had poor knowledge and awareness levels.

This finding was similar to that of a study conducted in Jeddah, Saudi Arabia, which also found a low level of knowledge regarding OA among the study population [9]. On the other hand, another study was conducted on the general population in Sudair, Saudi Arabia, found that the majority demonstrated good knowledge about OA [10].

OA is a degenerative disease that has many inflammatory markers and cytokines involved in its development [20]. A mixture of genetic susceptibility and poor lifestyle choices can lead to further progression of the disease [20-21]. When asked "Do you think osteoarthritis is caused by cold, damp weather?" and "Do you think it is developed by a microorganism?" only 25.7% and 45.4% of participants answered correctly, respectively, demonstrating a poor understanding of the pathophysiology of OA.

Women are more likely to develop OA [21-22], but only 35.8% of participants showed an awareness of the gender differences in OA, even though most participants were themselves female. Notably, 82.9% and 70.1% were aware that weight gain and aging, respectively, were risk factors. Genetics is another known risk factor [21], but less than half of the respondents (44.9%) were aware of this. Some respondents expressed concern that physical activity would make OA worse. The majority (67.8%) agreed that physiotherapy can result in significant reductions in OA symptoms [23].

In relation to OA management, non-steroidal anti-inflammatory drugs (NSAIDs) play a significant role in improving the symptoms of OA [24], but only 39.4% of the participants were aware of this. The mainstay of treatment for OA remains joint replacement [24], and half of the participants 50.4% knew that this will be the ultimate option.

Regarding sources of information about OA, the least reported source was from healthcare staff (8.9%). This was consistent with other studies [9]. It in turn demonstrated how important it is that healthcare professionals should increase efforts to improve awareness among their patients, families, and friends.

The major limitation of this study was the self-administered questionnaire method, which was suitable only for those who had access to the right technology, can read and were interested in participating. Further research is needed to explore population knowledge levels, as well as to determine what if any common misconceptions exist.

This research's clinical significance is increasing the awareness, knowledge gap, and misconceptions about OA and its risk factors. Moreover, due to the increase in the prevalence of OA, recent guidelines for OA management should be considered in Saudi Arabia. Weight loss for overweight or obese people, exercise, and walking aids for lower extremity osteoarthritis are among the most recent guidelines as non-pharmacological management for people with osteoarthritis [14]. Weight management has shown improvement in symptoms for people with weight-bearing osteoarthritis, such as the knee or hip [14,16].

Exercise, including resistance, aerobic, and mind-body exercise, are essential components of treatment, regardless of age, pain level, symptoms, and functional status [14]. Crutches are recommended for people with knee or hip OA, and orthosis have been suggested for hand OA [14,21]. Because of the high prevalence of OA in the GCC countries, it is important to use these recommendations for managing people with OA.

## Conclusions

The research indicated that OA awareness levels among the general population in Hail, Saudi Arabia, were unsatisfactory. Therefore, it is recommended that efforts be made to enhance awareness and knowledge levels through public education campaigns. In turn, this can help reduce risk factors and improve early detection of the disease. Hopefully, this will lead to overall improvements in the population’s lifestyle as well as reduce the socioeconomic burden of OA.

## Appendices

The Questionnaire:

Sociodemographic:

1.     Gender

2.     Age

3.     Educational level (uneducated, less than secondary, secondary, bachelors, higher education)

4.     Residence (city, rural)

General information’s:

1.     Have you been diagnosed with osteoarthritis? Yes, No

2.     Do you know how osteoarthritis develop?

3.     What is your source of information?

4.     Do you know anyone with osteoarthritis?

5.     Do you think weight gain is directly related to osteoarthritis?

(20 items quiz for assessing knowledge) (Yes, No, I don’t know) (correct answers= 1 score, incorrect = 0 score, I don’t know = 0 score)

1.     Do you think osteoarthritis is a chronic problem? 

2.     Do you think osteoarthritis is rare? 

3.     Do you think that different joints can be affected by osteoarthritis? 

4.     Do you think osteoarthritis is caused by cold, damp weather?

5.     Do you think it is developed by a microorganism? 

6.     Do you think pain is the only symptom of osteoarthritis? 

7.     Do you think stiffness is a symptom of osteoarthritis? 

8.     Do you think swelling is a sign of osteoarthritis? 

9.     Do you think osteoarthritis can lead to loss of joint movement? 

10.  Do you think there are genetic factors that can predispose parson to osteoarthritis? 

11.  Do you think aging is a risk factor for osteoarthritis? 

12.  Do you think men and women are equally affected by osteoarthritis? 

13.  Do you think physical examination and x-ray are used to diagnose osteoarthritis? 

14.  Do you think blood tests are used to diagnose osteoarthritis? 

15.  Do you think NSAIDs can improve osteoarthritis symptoms? 

16.  Do you think some forms of exercise like swimming is suitable for people with osteoarthritis? 

17.  Do you think acid-free diets are a proven treatment for osteoarthritis? 

18.  Do you think physiotherapy can cause a great improvement in the symptoms of osteoarthritis? 

19.  Do you think intra-articular injection by stem cell or hyaluronic acid is an effective modality for curing OA? 

20.  Do you think a joint replacement surgery will be the ultimate option to relieve the symptoms of osteoarthritis?
